# Characterization of the complete mitochondrial genome of *Dioszegia changbaiensis* (Tremellales: Bulleribasidiaceae) with phylogenetic implications

**DOI:** 10.1080/23802359.2021.1915197

**Published:** 2021-11-03

**Authors:** Maoling Tan, Qiangfeng Wang

**Affiliations:** a School of Food and Biological Engineering, Chengdu University, Chengdu, PR China; b Biotechnology and Nuclear Technology Research Institute, Sichuan Academy of Agricultural Sciences, Chengdu, PR China

**Keywords:** Yeast, mitochondrial genome, phylogenetic analysis, molecular marker

## Abstract

In this study, the complete mitochondrial genome of *Dioszegia changbaiensis* we sequenced and assembled by the next-generation sequencing. The complete mitochondrial genome of *Dioszegia changbaiensis* contained 22 protein-coding genes (PCG), two ribosomal RNA (*rRNA*) genes, and 22 transfer *RNA* (tRNA) genes. The total length of the *Dioszegia changbaiensis* mitochondrial genome is 34,853 bp, and the GC content of the mitochondrial genome is 41.88%. Phylogenetic analysis based on a combined mitochondrial gene dataset indicated that the mitochondrial genome of *Dioszegia changbaiensis* exhibited a close relationship with that of *Hannaella oryzae*.

The genus *Dioszegia* was described by Zsolt (1957). Since then, dozens of species have been described in the genus (Bai et al. 2002; Connell et al. 2010; Trochine et al. 2017). Species from the genus *Dioszegia* are distributed in a variety of ecological environments, such as on leaf surfaces, in plant roots and also in soil (Wang et al. 2003; Wang et al. 2008; Takashima et al. 2011). *Dioszegia* species was once moved to the genus *Cryptococcus* according to morphology (Takashima et al. 2011). With the progress of molecular techniques, the genus *Dioszegia* was separated from other genera as an independent genus (Takashima et al. 2001). Mitochondrial genomes have been widely used in the phylogeny of Basidiomycete species (Li, He, et al. 2020; Wang, Song, et al. 2020). The mitochondrial genome of *D. changbaiensis* reported here will promote the understanding of taxonomy and genetics of the *Dioszegia* genus.

The specimen (*Dioszegia changbaiensis*) was collected from Sichuan, China (102.53E; 31.25 N), and was stored in Culture Collection Center of Chengdu University (No. Dsp_na07). We sequenced and *de novo* assembled the complete mitochondrial genome of *Dioszegia changbaiensis* according to previous described methods (Li, Liao, et al. 2018; Li, Xiang, et al. 2019; Wang, Song, et al. 2020). Briefly, the total genomic DNA of *Dioszegia changbaiensis* was extracted using a Fungal DNA Kit (D3390-00, Omega Bio-Tek, Norcross, GA). And then we purified the genomic DNA using a Gel Extraction Kit (Omega Bio-Tek, Norcross, GA). The purified DNA was stored in Chengdu University (No. DNA_Dsp_na07). We constructed sequencing libraries of *Dioszegia changbaiensis* using a NEBNext^®^ Ultra^™^ II DNA Library Prep Kit (NEB, Beijing, China). We conducted whole genomic sequencing (WGS) of *Dioszegia changbaiensis* using the Illumina HiSeq 2500 Platform (Illumina, SanDiego, CA). The mitochondrial genome of *Dioszegia changbaiensis* was *de novo* assembled using SPAdes version 3.9.0 (Bankevich et al. 2012; Li, Ren, et al. 2020). The obtained mitochondrial genome of *Dioszegia changbaiensis* was annotated according to previous described methods (Li, Chen, et al. 2018; Li, Wang, et al. 2018; Wang, Jia, et al. 2020; Ye et al. 2020).

The complete mitochondrial genome of *Dioszegia changbaiensis* is 34,853 bp in length. The base compositions of the *Dioszegia changbaiensis* mitochondrial genome were as follows: A (28.49%), T (29.63%), G (20.27%), and C (21.61%). The complete mitochondrial genome of *Dioszegia changbaiensis* contains 22 protein-coding genes (PCGs), two ribosomal RNA genes (*rns* and *rnl*), and 22 transfer *RNA* (*tRNA*) genes. To investigate the phylogenetic status of the mitogenome of *Dioszegia changbaiensis* in Basidiomycota, we constructed a phylogenetic tree for 18 Basidiomycete species. *Rhizopogon salebrosus* from the Boletales order was set as outgroup (Li, Ren, et al. 2019a). The phylogenetic tree was constructed using the Bayesian analysis (BI) method based on the combined 14 core PCGs according to previous described methods (Li et al. 2019a, 2019b; Li, Yang, et al. 2020). As shown in the phylogenetic tree (Figure 1), the mitochondrial genome of *Dioszegia changbaiensis* exhibited a close relationship with that of *Hannaella oryzae* (MH732752)

.

**Figure 1.**
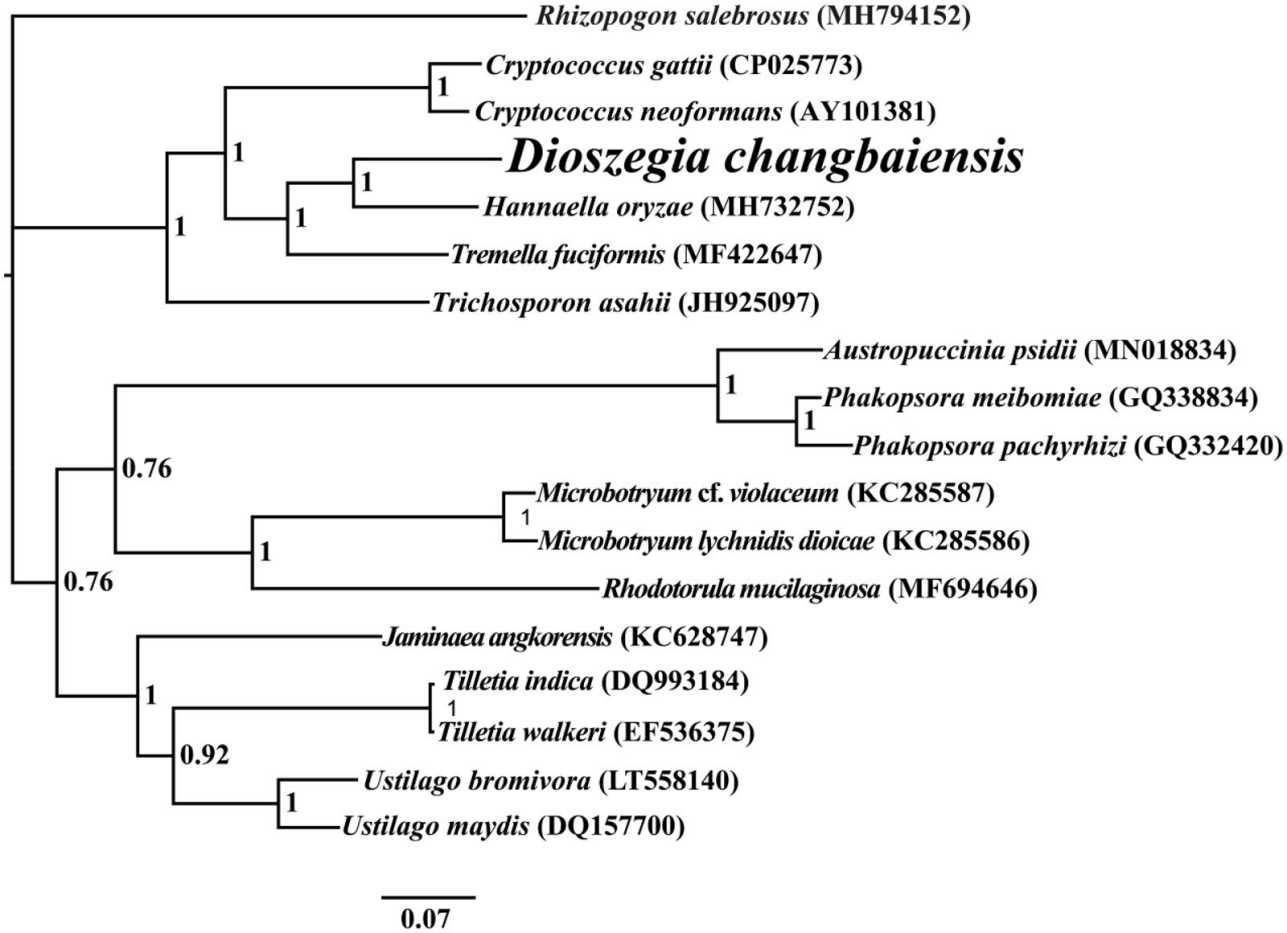
Bayesian phylogenetic analysis of 18 species based on the combined 14 core protein-coding genes. Accession numbers of mitochondrial sequences used in the phylogenetic analysis are listed in brackets after species.

## Data Availability

This mitogenome of *Dioszegia changbaiensis* was submitted to GenBank under the accession number of MT755637 (https://www.ncbi.nlm.nih.gov/nuccore/MT755637).
